# Synchronous liver and peritoneal metastases from colorectal cancer: Is cytoreductive surgery and hyperthermic intraperitoneal chemotherapy combined with liver resection a feasible option?

**DOI:** 10.3389/fsurg.2022.1006591

**Published:** 2022-12-15

**Authors:** Sara Di Carlo, Giuseppe Cavallaro, Francesca La Rovere, Valeria Usai, Leandro Siragusa, Paolo Izzo, Luciano Izzo, Alessia Fassari, Sara Izzo, Marzia Franceschilli, Piero Rossi, Sirvjo Dhimolea, Enrico Fiori, Simone Sibio

**Affiliations:** ^1^ Department of Surgery, Minimally Invasive Surgery Unit, University of Rome “Tor Vergata”, Rome, Italy; ^2^ Department of Surgery, Unit of Oncologic and Minimally Invasive Surgery, Sapienza University of Rome, Rome, Italy

**Keywords:** peritoneal metastases, cytoreductive surgery, liver resection, liver metastases, HIPEC, colorectal metastases.

## Abstract

**Background:**

Traditionally, synchronous liver resection (LR), cytoreductive surgery (CRS), and hyperthermic intraperitoneal chemotherapy for colorectal liver and peritoneal metastases have been contraindicated. Nowadays, clinical practice has promoted this aggressive treatment in selected cases. This study aimed to review surgical and survival results of an extensive surgical approach including CRS with hyperthermic intraperitoneal chemotherapy (HIPEC) and LR.

**Methods:**

PubMed, EMBASE, and Web of Science databases were matched to find the available literature on this topic. The search period was limited to 10 years (January 2010–January 2021). A threshold of case series of 10 patients or more was applied.

**Results:**

In the search period, out of 114 studies found about liver and peritoneal metastases from colorectal cancer, we found 18 papers matching the inclusion criteria. Higher morbidity and mortality were reported for patients who underwent such an extensive surgical approach when compared with patients who underwent only cytoreductive surgery and HIPEC. Also, survival rates seem worse in the former than in the latter.

**Conclusion:**

The role of combined surgical strategy in patients with synchronous liver and peritoneal metastases from colorectal cancer remains controversial. Survival rates and morbidity and mortality seem not in favor of this option. A more accurate selection of patients and more restrictive surgical indications could perhaps help improve results in this subgroup of patients with limited curative options.

## Introduction

Colorectal cancer (CRC) is a major health problem and is the leading cause of death in developed countries (1).

Metastatic diseases are present in approximately 20%–25% of patients with advanced CRC (2).

In patients with metastatic diseases from colorectal cancer, the liver and peritoneum are the most frequently affected sites; liver metastases (LM) are present in up to 55% of patients, while secondary peritoneal involvement (PM) affects up to 25% of patients (3–5).

Peritoneal carcinomatosis is considered a negative prognostic factor in metastatic colorectal cancer (6). Peritoneal carcinomatosis occurs when the tumor invades the bowel serosa, allowing malignant cells to shed and circulate through the peritoneal fluid. During surgery, iatrogenic manipulation may lead to tumor cells seeding within the peritoneal cavity; these tumor cells implant in the peritoneal microenvironment with blood vessels and lymphatics. Due to gravity and physiologic peritoneal fluid circulation, anatomical sites of the peritoneum that are most frequently affected include the upper abdominal regions such as the subphrenic regions, the lesser sac, bowel surfaces, mesentery, and in the pelvis. Tumor cell implantation leads to tumor plaque formation that may then involve extending to peritoneal surfaces (7, 8). The National Comprehensive Cancer Network (NCCN) guidelines recommend, in high-volume centers and for patients with limited peritoneal metastases [i.e., peritoneal cancer index—peritoneal cancer index (PCI) not more than 16–20, depending on different experiences], cytoreductive surgery (CRS) in association with hyperthermic intraperitoneal chemotherapy (HIPEC) (9).

Metastatic spread from the primary tumor to the liver occurs through hematogenous dissemination. The production of tumor growth factors induces the secretion of vascular endothelial growth factor that stimulates the generation of new endothelial cells through angiogenesis. Malignant cell dissemination happens from microscopic vessels to the portal venous system and liver sinusoids, which represent the suitable microenvironment for tumor growth (10).

Oligometastatic diseases with combined hepatic and peritoneal metastatic spread affect approximately 8% of those with CRC (6), especially the presence of peritoneal metastases associated with shorter overall survival (OS) (11). The prognosis of patients with isolated LM or isolated peritoneal metastases (PM) has improved with the combination of systemic chemotherapy and complete resection, yielding a 5-year overall survival rate of 40%–50% (12, 13). CRS with intraperitoneal chemotherapy, including HIPEC, has been considered a potentially curative treatment for PM of CRC, reaching a median OS of 31 months and up to 41 months in highly selected patients (14–16).

The best strategy to treat advanced colorectal cancer with synchronous peritoneal and liver metastases (PMLM) is unclear; in the past, this was considered a terminal condition, and these patients were referred to palliative care with systemic chemotherapy with a median survival of 12–24 months (17).

A change in the trend started in 2008 when patients with CRC with up to three or fewer small resectable parenchymal hepatic metastases, good performance status, and no major comorbidities could be considered as candidates for complete R0 resection of all tumors with CRS, liver resection (LR), and hyperthermic intraperitoneal chemotherapy (HIPEC) (18).

In recent years, smaller pilot series have shown, in highly selected patients, excellent median survival beyond 40 months in resections of simultaneous liver and peritoneal metastases with CRS plus HIPEC (16, 19–23). However, to date, no standard management has been established.

Moreover, the resectability rate in patients with unresectable or multiple hepatic metastases can be increased by approaching these cases with advanced procedures such as portal vein embolization or two-stage hepatectomy (24).

Optimizing patient selection with good performance status or with minimal comorbidity and accurate perioperative management is crucial to maximizing patient outcomes while minimizing morbidity and mortality. Variations in outcomes depend on the severity of the disease represented by the PCI, tumor differentiation, histologic findings, liver extension, and the completeness of cytoreduction (25, 26). Currently, centers demonstrate large heterogeneity in whether combining CRS–HIPEC with liver resection can offer beneficial results.

Given the contradicting data and the lack of standardized management for patients with simultaneous peritoneal and hepatic metastases from CRC, a thorough evaluation of the current literature is warranted to guide the correct strategy for these patients.

This study aimed to review surgical and survival results of an extensive surgical approach including CRS + HIPEC combined with LR in patients affected by peritoneal and hepatic metastases from CRC.

## Methods

PubMed, EMBASE, Cochrane, and Web of Science databases were matched to find the available literature on this topic. The search period was limited to 10 years (2010–2021) to consider only up-to-date experiences in this relatively recent field of integrated treatments. Search terms including synonyms and keywords such as “metastatic colorectal cancer, HIPEC, intraperitoneal chemotherapy, liver metastases, liver resection, hepatectomy, peritoneal carcinomatosis, and peritoneal metastases” were used. Case reports, case series analyzing fewer than 10 patients, and duplicate articles were excluded. Two reviewers screened all potentially relevant titles and abstracts, selecting papers that described patients treated with CRS–HIPEC who had peritoneal and liver metastases. English-language articles were eligible for inclusion if they specified types of studies [randomized control studies (RCTs), cohort studies, case–control studies, and cross-sectional studies], types of participants (patients with colorectal cancer metastasized to the liver or peritoneum), and types of treatments (both CRS and HIPEC). The review excluded letters to the editor, case reports, reviews, and meta-analyses. Data were collected from the included studies. Patients were divided into two groups: a group of patients with PM only and a group of patients with PMLM. The primary endpoints were OS and disease-free survival (DFS) calculated from the date of CRS–HIPEC. The secondary endpoints were perioperative outcomes including morbidity and mortality. Major morbidity was defined as the presence of a complication classified as Clavien–Dindo grade 3 or higher. Data on length of stay, operative time, PCI, pre- and postoperative chemotherapy, and follow-up period were also recorded (Tables 1 and 2).

**Table 1:** Surgical outcomes of available literature experiences.

	Study period	Patients		Operative time (min)		PO mortality (%)		Major PO morbidity (%)		Hospitalization (days)		Median Follow-up (months)	
		LM + PM	PM	LM + PM	PM	LM + PM	PM	LM + PM	PM	LM + PM	PM	LM + PM	PM
Allard 2013	1985–2010	30	NR	NR	NR	0	0	16.6	NR	NR	NR	63	NR
Blackham 2014	1991–2010	179	93	300	540	3.9	5.4	21	23	6	9	58	89
Alzahrani 2015	2003–2014	36	42	366	480	NR	NR	38.9	31	21.8	23.7	21.9	21.5
Randle 2015	1991–2013	32	201	528	510	6.5	2.8	18.5	22.5	13.6	14.2	75	120
Delhorne 2015	2007–2011	9	18	NR	NR	0	5	44.4	11.1	22	20.5	14	18
Berger 2016	2007–2014	103	166	379.3	316.9	5.8	6.7	24.3	18.1	8	6	18.2	18.2
Larimier 2016	1999–2011	22	36	586	456	1.9	11.1	54.5	38.8	22	19	60	60
Navez 2016	2007–2015	25	52	NR	NR	0	4	32	15.4	19	13	25.5	34.2
Saxena 2016	1996–2016	132	803	522	522	2.2	1.7	40.1	41.9	28	28	36	36
Morales Soriano 2017	2010–2015	16	45	456	420	0	4.4	56.3	26.6	23.1	14.4	20	20
Downs-Canner 2017	2005–2013	32	173	520.9	470.8	3.5	1.2	32.3	16.7	16	17.2	60.9	56.8
Mouw 2018	2005–2016	20	23	NR	NR	5	0	40	13.4	12.3	9.8	NR	NR
Cloyd 2018	2005–2016	100	1068	520.7	454.6	3	1.4	47	27.4	16.7	11.1	NR	NR
Jean 2019	2014–2018	22	NR	684	NR	4.5	NR	22.7	NR	25.6	NR	34	NR
Pinto 2019	2007–2016	33	76	420	420	0	0	42.4	39.4	28	25	30	30
Horvath 2019	2006–2016	37	NR	431	NR	0	NR	42	NR	9	NR	23	NR
Lo Dico 2020	1993–2017	437	NR	NR	NR	3.2	NR	40.2	NR	22.9	NR	60	NR
Lee 2020	2000–2017	83	575	504	429	NR	NR	81	60	NR	NR	23	23

LM, liver metastases; PO, post-operative; NR, not reported.

**Table 2:** Survival data and follow-up.

	Median PCI		Neoadjuvant therapy (%)		Adjuvant therapy (%)		Median OS		Median DFS		Recurrence rate (%)		HIPEC	CCR	
	LM + PM	PM	LM + PM	PM	LM + PM	PM	LM + PM	PM	LM + PM	PM	LM + PM	PM	LM + PM	LM + PM	PM
Allard 2013	2	NR	83,3	NR	100	NR	42	NR	NR	NR	83	NR	NR	NR	NR
Blackham 2014	N	NR	39	65	62	41	45.7	33.6	17.5	17.3	NR	NR	MMC	95	51.6
Alzahrani 2015	7	12	92	41	92	81	24.4	45.5	8.5	17.7	86	71	MMC/OX	97	93
Randle 2015	NR	NR	100	100	NR	NR	21.2	33.6	6.8	12	64.7	53.3	NR	42.2	45.7
Delhorne 2015	19	9	100	100	11	11	27.6	39.1	6.2	12	89	95	MMC or OX	100	100
Berger 2016	17.5	10	58.2	56.6	NR	NR	45.1	73.5	17.3	13.2	NR	NR	MMC	83.5	81.8
Lorimier 2016	15	10.5	86.2	86.2	90.9	83.3	36.1	25.2	9.5	12.6	NR	NR	MMC or LOHP	86.4	69.4
Navez 2016	10	6	90.5	57.7	52.4	87.5	27.5	59.2	6.7	18.4	81	NR	OX or MMC	100	100
Saxena 2016	NR	NR	NR	NR	NR	NR	32.3	30.5	14	14	NR	NR	NR	78	64.2
Morales Soriano 2017	10.6	9.9	81.3	73.3	NR	NR	36	33	12	12	11.4	NR	OX or MMC	100	100
Downs-Canner 2017	13.7	11.2	97	NR	69	63	13	20.5	9.9	7.6	22.6	NR	MMC	100	100
Mouw 2018	NR	NR	NR	NR	NR	NR	NR	NR	NR	NR	60	65.2	MMC/OX	100	82.61
Cloyd 2018	NR	NR	16	8.8	NR	NR	NR	NR	NR	NR	NR	NR	NR	NR	NR
Jeon 2019	13	NR	90.9	NR	95.5	NR	16.7	NR	7.1	NR	81.8	NR	MMC	100	NR
Pinto 2019	9	6	100	88	54.5	36.8	31	65	21	24	66.6	51.3	OX	100	98.6
Horvath 2019	14	NR	78	NR	NR	NR	22	NR	59.5	NR	29.7	NR	Cisplatin/MMC/OX	100	NR
Lo Dico 2020	9.8	NR	79.8	NR	60.5	NR	44.8	NR	17.8	NR	77.9	NR	OX	100	NR
Lee 2020	12.8	12.8	59	44	NR	NR	20	25	NR	NR	NR	NR	NR	NR	NR

PCI, peritoneal cancer index; OS, overall survival; DFS, disease-free survival; HIPEC, hyperthermic intraperitoneal chemotherapy; LM, liver metastases; MMC, mitomicin C; OX, oxaliplatin; LOHP, oxaliplatin.

## Results

**Figure 1:**
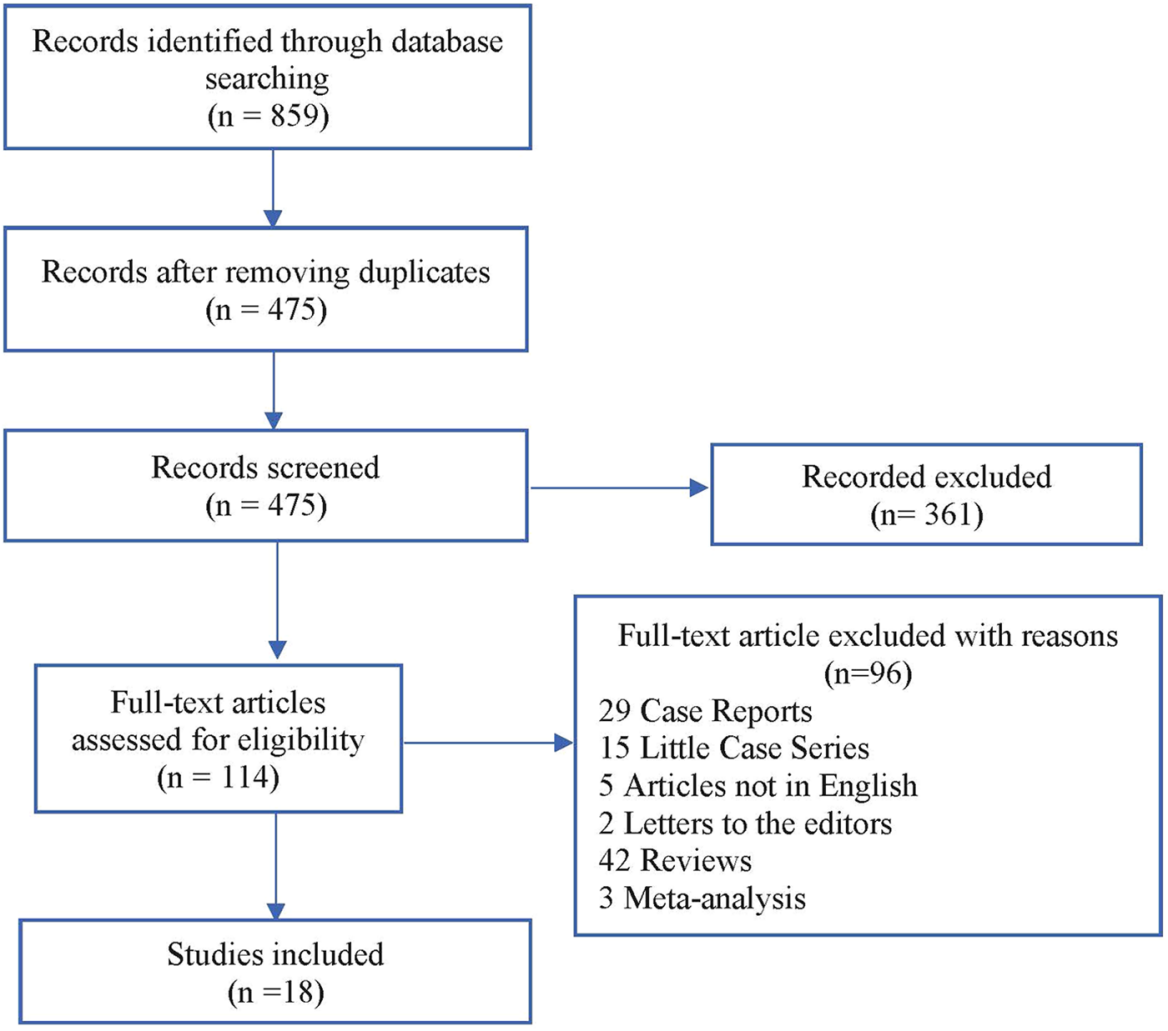
Flow chart (PRISMA guidelines) of the reviewed studies.

Our literature search identified 859 studies. After removing duplicates, 361 of the 475 remaining studies were excluded based on title and abstract assessment. Exclusion criteria are as follows: studies describing only peritoneal metastases patients, studies describing only colorectal liver metastases patients, articles reporting multiple types of malignancies, where differentiation between patients with colorectal cancer and those with other types of tumors was not possible, articles in which survival outcomes have not been clearly reported, article that failed to extract survival data comparing the peritoneal metastases + liver metastases group with the peritoneal metastases alone group, articles that failed to retrieve peritoneal metastases in combination with colorectal liver metastases data, or studies about debulking surgery alone or in combination with systemic chemotherapy. Out of 114 remaining studies, we found only 18 studies in which data on procedures and outcomes could be completely retrieved. A flow diagram of the literature search procedure according to the PRISMA guidelines is shown in Figure 1.

All 18 studies included in the review were published during the study period. In total, 4,719 patients were included in the study. Of these, 1,348 patients presented with synchronous PC + LM and had been treated with liver resection (or alternative therapy such as radiofrequency ablation—RFA) in combination with CRS and HIPEC. The remaining 3,371 patients presented with isolated PC and had been treated with CRS and HIPEC. In most of the studies, the PCI was comparable, and in all cases, it was below 20, which corresponds with clinical guidelines (Table 2). With the exception of the studies by Pinto et al. (28), Lo Dico et al. (40), and Jeon et al. (35), the studies in this review presented patients treated in a one-step procedure with CRS–HIPEC and liver resection/ablation performed during the same surgical procedure. Only a few studies reported the number of liver lesions. In most cases, liver resection was limited to small resection and RFA. Details of the liver treatment are presented in Table 3.

**Table 3:** Extent of liver disease and types of liver treatments.

Study	Extent of liver disease (No. of lesions)	LM treatment
Allard 2013	1: 391 pts	Resection
	2:376 pts	
	3 or more: 397 pts	
Blackham 2014	Mean 1.9	Resection and or RFA
Alzahrani 2015	<3: 25 pts	Resection
	>3: 11 pts	
Randle 2015	Not recorded	NR
Delhorne 2015	Median 1	Resection and or RFA
Berger 2016	Not recorded	Resection
Lorimier 2016	Mean 1.9	Resection and or RFA
Navez 2016	<3	Resection and or RFA
Saxena 2016	1:34 pts	NR
	2–3: 30 pts	
	4 or more: 6	
Morales Soriano 2017	Mean 1.2	Resection and/or RFA
Downs-Canner 2017	1:16 pts	Resection and/or RFA
	2: 7 pts	
	3 or more: 7	
Mouw 2018	Not recorded	Resection
Cloyd 2018	Not recorded	Resection
Jeon 2019	Mean 3	Resection and/or RFA
Pinto 2019	Not recorded	Resection and/or RFA
Horvath 2019	1–2: 24 pts	Resection
	>2: 4 pts	
Lo Dico 2020	Median: 1	Resection
Lee 2020	Not recorded	Resection

RFA, radiofrequency ablation; LM, liver metastases.

## Discussion

This review shows that combined treatment of peritoneal and hepatic metastases for selected patients is feasible, resulting in a mean overall survival of 30 months. Combined CRS–HIPEC and liver resection can be an alternative for patients with limited diseases, leading to an improvement in terms of survival compared to patients who could receive only systemic therapy (42, 44). Despite the feasibility and safety of the combined LR and CRS–HIPEC in metastatic CRC reported from several studies (20, 22, 23, 27, 33, 36, 37, 39, 45), data on the matter show conflicting results, with updated studies and meta-analyses demonstrating evidence to the contrary (5, 21, 32, 34, 46). Razenberg et al. (47) reported a significantly lower median OS in patients with concomitant PC + LM treated with palliative chemotherapy compared to the patients treated with CRS and HIPEC (12.5 vs. 23.1 months). However, there could be a biased selection in interpreting this result as no data regarding the two groups (dissemination of the disease, history prior to treatment, and general conditions of the patients) were available. Lo Dico et al. (39), in their multicenter study, showed that extended surgical management with curative resection plus HIPEC in selected patients with PM + LM is feasible with acceptable morbidity and mortality rates (31% and 4%, respectively) and a better OS. These results are probably associated with a better selection of patients and with the choice of performing the combined procedure only if a minor LR was required. In fact, the study suggested performing a liver-first approach in the case of a two-step procedure and when a minor resection was not feasible. Our primary aim was to review the surgical and survival results of an extensive surgical approach including CRS + HIPEC and LR. Our updated literature review found worse perioperative outcomes (40% vs. 25%) among patients undergoing synchronous LR and CRS–HIPEC compared to the patients undergoing CRS–HIPEC alone. However, no data were available to clarify the risk factors to determine the difference in morbidity. Our results are in line with the findings of Cloyd et al. (35), who described that concomitant LR and CRS/HIPEC were associated with an increased number of postoperative complications and increased readmission compared to patients undergoing CRS/HIPEC alone. However, contradictory results of single-institution studies reporting no difference in postoperative morbidity have been published (3, 23, 27, 33). Lorimier et al. (27), in their monocentric retrospective study, showed better median OS in the PCLM group compared to the PC group only (36 and 25 months, respectively) but without significant statistical difference and with the same OS rate at 5 years (>40%). However, patients in the PCLM group had more hepatic and peritoneal recurrence than those in the PC group. Mortality linked to the surgical procedure was 6.8%, and global morbidity was 38%, without a significant difference between the two groups. In accordance with previous publications, the major postoperative complications occurred more frequently in patients with a PCI >20. Maggiori et al. also described a morbidity of 51% and mortality of 8% for patients undergoing the combined procedure, but almost half of the patients underwent major hepatectomy (48). Delhorme et al. (20) confirmed a significant morbidity rate (44%) when concomitant HIPEC and LS were performed compared with HIPEC alone (11%). Navez et al. (23) described a morbidity rate of 32% when the combined procedure was performed and a median OS of 27.5 months (25). Major postoperative complications were higher in the study by Down-Canner et al. (34) as well (32% vs. 17%). Furthermore, most studies showed a trend toward a shorter median survival time in the PC + LM group and the median OS reported was 29 months. These adverse clinical outcomes should be considered when selecting patients for such aggressive treatment, given that it may provide minimal benefit in terms of prognosis. Nevertheless, other additional factors should be considered in the selection of the patients. For example, survival is also associated with PCI, which is used to evaluate disease extent in peritoneal surface malignancies. Low PCI and the completeness of cytoreduction (CC-0 or -1) were demonstrably associated with a survival benefit with an inverse linear relationship present between PCI and OS; PCI is in fact recognized as an independent prognostic indicator in patients with metastatic peritoneal disease (49). Maggiori et al. (48) reported a median OS of 40 months in patients with a PCI <12 and ≤2 LM, and a higher PCI and more LM were associated with a lower OS (17). Alzahrani et al. showed that the median survival for patients with PCI ≤ 7 and ≤ 3 LM was longer than those with a PCI > 7 and >3 LM (31). Soriano et al. recommended not to perform completeness of cytoreduction rate (CCR) + HIPEC in patients with a PC index higher than 18 points because of its elevated morbidity and poor survival and limited the simultaneous hepatic and peritoneal resection to patients with three or fewer liver lesions (41). Further research is necessary to determine the prognostic effect of these two variables and the relationship with other variables such as tumor histology, performance status, and lymph node metastasis. In a recent review, Lo Dico et al. reviewed all the available major experiences in the combined treatment of liver and peritoneal metastases from colorectal cancer, and their results suggested that patients with limited peritoneal disease (mean PCI of all the reviewed studies was 9.8) and those who need minor liver resections (defined as fewer than three hepatic segments) are the most likely to have better prognostic outcomes (39).

In the past, the presence of synchronous liver and peritoneal metastatic disease was considered a contraindication to surgical resection, and palliative chemotherapy was considered the only possible option (21). Systemic chemotherapy can improve the prognosis, achieving a median OS of 12–16 months (43, 50). Compared to classical chemotherapy regimens, the FOLFOXIRI regimen has shown better in metastatic CRC patients (51). By performing CRS with hyperthermic intraperitoneal chemotherapy, median OS can be brought up to 31–40 months with complete macroscopic resection, which could be increased even more through accurate patient selection (20). Regarding HIPEC role and toxicity, a recent prospective randomized multicenter phase III French trial (PRODIGE 7) has raised concerns about the benefits of adding HIPEC to CRS on survival in patients who underwent CRS + HIPEC compared to those who underwent CRS only (39). Regarding the results of our review, only a few papers report LR + CRS without HIPEC, and mostly, this happens when a minimal peritoneal disease is discovered accidentally and thus resected (29, 52). In larger experiences, the association of HIPEC to CRS correlates with a survival advantage and only a little increase in morbidity. HIPEC should be avoided only in cases where the expected increase in morbidity could be high (for example, patients with multiple comorbidities, renal, hepatic, or bone marrow failure, representing common contraindications to HIPEC) (39).

Certainly, drugs, regimens, and intraperitoneal (IP) perfusion duration influence results. Currently, two regimens are widely used: open-abdomen oxaliplatin ± irinotecan with concurrent intravenous 5-fluorouracil and folinic acid and open- or close-abdomen mitomycin-C, alone or in combination with other drugs (52). In these specific settings of patients, IP regimens with oxaliplatin seem to provide the best improvement in outcomes. Whether this improvement depends on the use of a specific drug or the different duration of IP perfusion (30 vs. 90 min) remains debatable (53).

However, in the reported experiences considered for this review, no increased toxicity of chemotherapeutic agents has been observed in patients who underwent LR compared to those who did not. Pinto et al. reported a median OS of 31 months for patients who underwent HIPEC + LR and received neoadjuvant chemotherapy, highlighting how the response or nonprogression during neoadjuvant treatment can be beneficial in selecting patients. He also proposed a two-step procedure for patients with bilobar metastases to avoid major hepatic resection during HIPEC, reducing postoperative morbidity and mortality rates (28). In fact, in the presence of hepatic metastases, the resectability rate can be increased by several surgical techniques, such as two-stage hepatectomy or portal vein embolization, even in patients with initially unresectable, multiple secondary diseases. LM may require only minor liver surgery procedures, usually performed at the same time as CRS and HIPEC, or it may require complex liver resection surgery that could be performed by two-step procedures; hence, HM management could be adapted depending on the extension of the metastatic disease and even the need for aggressive liver surgery such as major hepatectomy that could be performed in the simultaneous, delayed, and liver-first approach (14). Commonly, liver surgery is limited to minor resections in most of the experiences because cytoreductive surgery associated with major liver surgery, such as two-stage hepatectomy followed by HIPEC, seems to be correlated to unacceptable morbidity rates in the few papers that considered this approach (28–30). Other major liver procedures such as associating liver partition and portal vein ligation for staged hepatectomy (ALPPS) are not described in the papers considered for this review. This review shows that combined integrated local treatment of peritoneal and hepatic metastases for selected patients is feasible, although its outcomes remain controversial. The survival rates of these patients suggest an advantage compared with patients who only received systemic chemotherapy. On the other hand, major morbidity rates seem to be worsened by the association of two major surgical procedures like LR and CRS + HIPEC. From this point of view, a key role is played by the extension of hepatic and peritoneal surgical resections that should represent a cornerstone in the preoperative evaluation of these patients, as more aggressive surgical procedures have been demonstrated to link with a higher rate of postoperative complications, as clearly reported by major experiences in the field (40). Aside from the surgical extension, specific organs resection also seems to be linked to the morbidity rate such as rectal resection or organ resections associated with upper quadrant peritonectomy (i.e., resection of the diaphragm, spleen, or pancreas) (33, 36, 54). As operative and patient factors both contribute to morbidity and mortality, additional factors that should be considered are the number of hepatic metastases, liver function tests, low or intermediate PCI scores, types of drugs and perfusion's duration of intraperitoneal chemotherapy, patient's characteristics such as age, performance status, and comorbidities, and tumor characteristics including tumor histology and grading (advanced tumors or signet ring cell histology), neoadjuvant therapy, and response to systemic chemotherapy (RECIST criteria). The incidence of major complications represents one of the most determinant factors limiting the results of this combined approach and the most relevant in worsening prognosis. Another significant factor impacting morbidity and hence prognosis is the number of liver metastases. An attempt to preoperatively estimate the expected survival after the combined procedure has been proposed by Elias et al. by the development of a nomogram including criteria such as the number of liver metastases, PCI, and type of surgery (CRS/HIPEC alone, LR alone, or concomitant LR and CRS/HIPEC) (20, 55). Patient selection and risk stratification may also be carried out by the use of risk scores in which an increased number of factors detected has been associated with decreased OS; factors proposed to assess the risk score are patient's age, primary tumor histology, number of liver lesions (single vs. multiple), and pathways of recurrence (38, 56–58). The median follow-up in our review was 32 months (20–63 months), and recurrence rates were respectively 81% and 71% in the PM + LM group and the PM group regardless of the additional use of neoadjuvant or adjuvant therapy. The incidence of postoperative mortality was 2.6% in the PM + LM group and 2.8% in the PM group. No studies showed a significant difference in postoperative mortality between the two groups. To date, neither patient selection nor patient management criteria have been standardized for combined treatment; considering the aforementioned survival rates and morbidity and mortality data, extensive surgical approaches including CRS and hepatic LR should not be defined as safe and risk-free, as some studies previously reported. Nevertheless, accurate patient selection and an individualized preoperative decision-making process should be considered fundamental steps in the initial management of patients selected for combined treatment (59, 60).

## Conclusion

The role of combined surgical strategy (CRS + HIPEC and LR) in patients with synchronous liver and peritoneal metastases from colorectal cancer remains controversial. Survival rates and morbidity and mortality seem not in favor of this option. A strict and homogeneous selection of patients and a “tailored” surgical strategy (one-step vs. two-step liver surgery, extent of cytoreduction, and increasing use of laparoscopic techniques) (61–64) are mandatory to obtain the best results without increasing morbidity, and it would perhaps help improve the misleading results in this subgroup of patients with limited curative options.

**Table:** 

Core statements
	The role of combined surgical strategy (cytoreductive surgery with HIPEC and liver resection) in patients with synchronous peritoneal and liver metastases from colorectal cancer is promising but controversial.
	Advantages in survival rates from the combined procedure seem encouraging, but high morbidity rates still limit the widespread of this approach.
	Homogeneous patient selection criteria and preoperative decision-making processes are still lacking, even if some attempts in recent years have been made to standardize procedures and indications.
	More efforts are needed to clarify which patients could really benefit from this complex combined strategy and which risk rates could be considered acceptable.
